# Mechanism of Action of Dihydroquercetin in the Prevention and Therapy of Experimental Liver Injury

**DOI:** 10.3390/molecules29153537

**Published:** 2024-07-27

**Authors:** Hewei Wei, Ting Zhao, Xinglong Liu, Qiteng Ding, Junran Yang, Xiaoyu Bi, Zhiqiang Cheng, Chuanbo Ding, Wencong Liu

**Affiliations:** 1 College of Traditional Chinese Medicine, Jilin Agricultural University, Changchun 130118, China; www03211@126.com (H.W.); ding152778@163.com (Q.D.); junran231201@163.com (J.Y.); bxy123163@163.com (X.B.); czq5974@163.com (Z.C.); 2 School of Food and Pharmaceutical Engineering, Wuzhou University, Wuzhou 543002, China; lyguiwandingding@163.com (T.Z.); xinglongliu1221@126.com (X.L.); 3 College of Traditional Chinese Medicine, Jilin Agriculture Science and Technology College, Jilin 132101, China

**Keywords:** dihydroquercetin, liver injury, biological availability, mechanism

## Abstract

Liver disease is a global health problem that affects the well-being of tens of thousands of people. Dihydroquercetin (DHQ) is a flavonoid compound derived from various plants. Furthermore, DHQ has shown excellent activity in the prevention and treatment of liver injury, such as the inhibition of hepatocellular carcinoma cell proliferation after administration, the normalization of oxidative indices (like SOD, GSH) in this tissue, and the down-regulation of pro-inflammatory molecules (such as IL-6 and TNF-α). DHQ also exerts its therapeutic effects by affecting molecular pathways such as NF-κB and Nrf2. This paper discusses the latest research progress of DHQ in the treatment of various liver diseases (including viral liver injury, drug liver injury, alcoholic liver injury, non-alcoholic liver injury, fatty liver injury, and immune liver injury). It explores how to optimize the application of DHQ to improve its effectiveness in treating liver diseases, which is valuable for preparing potential therapeutic drugs for human liver diseases in conjunction with DHQ.

## 1. Introduction

The liver is an indispensable organ for the maintenance of life activities of the organism and is the leading site for anabolism, catabolism, and energy metabolism [1]. Liver injury is a condition in which hepatocyte damage to the liver occurs due to various causes, which in turn affects normal liver function. Globally, about 2 million people die from liver disease every year (data provided by the Institute for Health Metrics and Evaluation, University of Washington and Department of Surgery, University of Texas Southwestern, 2014) [2,3]. Liver injury occurs due to several factors, the most common of which are infectious, including hepatitis A, B, C, D, and E [4,5]. In addition, other factors such as drugs, alcohol, fat, and autoimmune can trigger liver injury. Liver injury can be categorized into viral liver injury, drug liver injury, alcoholic liver injury, fatty liver injury, and autoimmune liver injury. Liver injury, if not controlled in a timely and reasonable manner, can lead to the occurrence of serious diseases such as cirrhosis and even liver cancer [6,7]. Therefore, it is crucial to fully understand liver diseases and find safe and effective treatment methods to prevent their occurrence and development.

**Figure 1:**
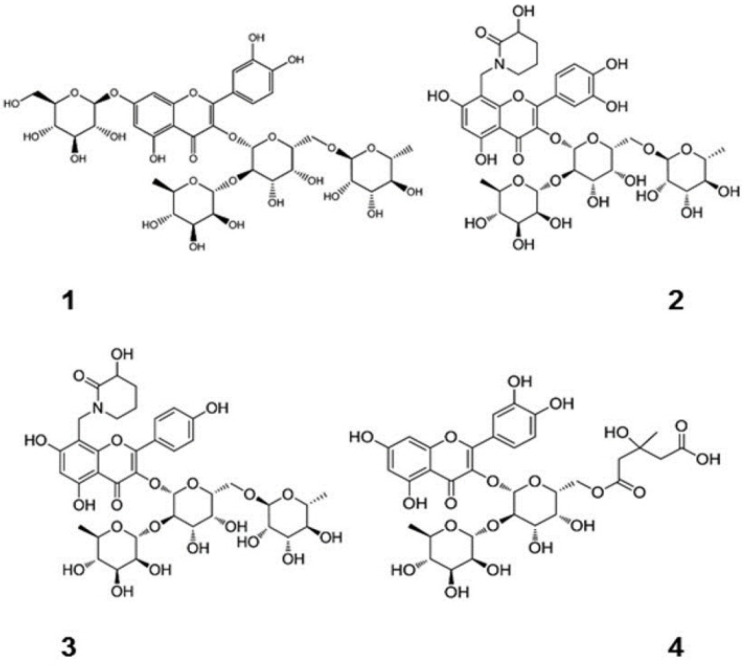

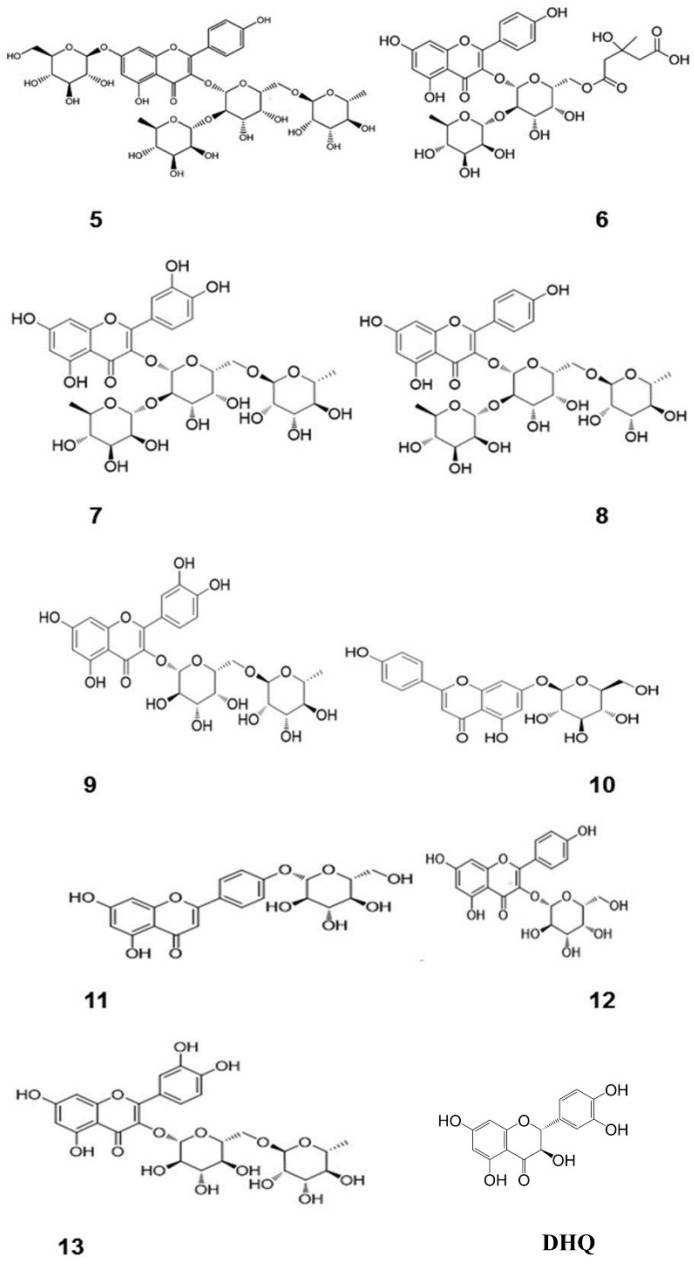
Chemical structures of flavonoids containing different sugar groups and DHQ [12]. (These figures were reprinted with permission).

In recent years, natural medicinal plants have shown promising potential in protecting against liver injury, with flavonoids standing out as a particularly noteworthy area of research [8,9,10]. Pardede et al. [11] demonstrated the hepatoprotective effect of the active compound rutin on Tert-butyl Hydroperoxide (*t*BHP)-induced Hep G2. Kondeva et al. [12] found that flavonoids extracted from many species of the genus *Astragalus* effectively inhibited the release of lactate dehydrogenase (LDH) and reduced the production of malondialdehyde (MDA), thereby preventing liver injury. The effects of flavonoid structures on hepatotoxicity were analyzed using a QSAR model with LDH activity and MDA production as indicators. The analysis results showed that the presence of aromatic hydroxyl groups in flavonoids increased the number of non-hydrogen bonded aromatic carbon atoms attached to them. The negative regression coefficients in the model reveal that the higher the number of such aromatic carbon atoms, the lower the toxicity of the compounds to the liver. At the same time, the lipophilicity of flavonoids increases with the decrease in the number of hexose units. The positive regression coefficients in the model confirm the idea that the more lipophilic a compound is, the more toxic it is to the liver. In summary, flavonoids may have this hepatoprotective property due to the variety of functional groups contained in their glycosidic structure or glycosyl portion, and these different functional groups confer specific bioactivities (Figure 1). Dihydroquercetin (DHQ), as a natural and highly active flavonoid, is more widely distributed in plants, including tiger balm, mulberry, grape, elderberry, mistletoe bark, and wood bean root. Nevertheless, it is mainly derived from the roots of larch in alpine regions [13]. Like other flavonoids, DHQ possesses a wide range of bioactivities. It can be used to treat many causes of liver disease as well as anti-inflammatory [14,15], antiviral [16], antidiabetic [17], and other factors. Therefore, it is widely used in medicine and other fields and has a large potential for development.

In this review, scientific databases such as X-MOL, PubMed, Web of Science, and Google Scholar were searched from 1990 to the present to find out the online scientific literature on the preventive and curative effects of DHQ on liver injury and the improvement of DHQ bioavailability. The keywords searched were dihydroquercetin, taxifolin, liver injury, and biological availability. Based on this review, this paper summarizes the mechanism of action of DHQ in liver injury and the research progress in recent years in terms of five aspects: pharmacological liver injury, alcoholic liver injury, non-alcoholic liver injury, fatty liver injury, and immune liver injury. We also reviewed the technical means to improve the biological availability of DHQ to provide theoretical support for the broad application of DHQ in the future.

## 2. Mechanism of Action of DHQ in Protection against Liver Injury

### 2.1. Ameliorative Effects on Drug-Induced Liver Injury

Drug-Induced Liver Injury (DILI) is a condition in which liver function is impaired during drug use due to the drug itself, its metabolites, or an individual’s abnormal sensitivity and decreased tolerance to the drug, also known as drug-induced liver disease [18]. This injury can present with acute or chronic hepatitis symptoms, and liver function usually resolves spontaneously after the discontinuation of the drug in mild cases. Regardless, in severe cases, DILI can be life-threatening and requires urgent treatment [19]. It is noteworthy that DILI does not discriminate between populations and may occur in people with otherwise healthy livers or affect people with severe disease. DILI is complex and difficult to predict. Therefore, monitoring changes in liver function during drug therapy and recognizing and managing drug-related liver injury promptly are essential for patient safety.

#### 2.1.1. Ameliorative Effects of Acetaminophen (APAP)-Induced Liver Injury

Acetaminophen (APAP) has been one of the widely used antipyretic and analgesic drugs worldwide since 1950 [20,21]. The drug is generally considered safe at the recommended dose (no more than 4 g per day) [22]. However, if this dosage limit is exceeded, an overdose can cause severe liver damage and may even progress to acute liver failure (ALF), a potential risk that should not be ignored [23,24,25]. Research has indicated that the metabolic processes of drugs and their effects on diverse intracellular signaling pathways play a central role in the mechanism of drug-induced hepatotoxicity, especially perturbations involving mitochondrial function, which are of particular importance [23]. When widespread necrosis occurs in the liver, the ensuing sterile inflammatory response is a crucial component of the body’s attempt to restore and repair damaged tissue. However, this inflammatory response acts as a double-edged sword: on the one hand, it is an integral part of tissue repair; on the other hand, if it is not regulated correctly, it may exacerbate the pre-existing damage and result in a more complex pathological state [26]. This is a controversial area. Despite this, a more profound comprehension of these inflammatory pathways is expected to facilitate the discovery of new therapeutic targets, which are especially important during the transition from the injury phase to the regeneration phase.

Chen et al. [27] first investigated the protective effect of DHQ against APAP-induced liver injury in a mouse model. Mice were injected intraperitoneally with a certain amount of APAP to establish the model. One hour later, they were treated with different concentrations (0, 20, 40 mg/kg) of DHQ. The high dose of DHQ was able to effectively inhibit the mRNA expression of the TLR4 gene and down-regulate the mRNA levels of pro-inflammatory factors TNF-α and IL-6. It significantly restored the reduction of antioxidant enzyme activities induced by APAP at both the mRNA and protein levels. The expression of Bcl-2 and pro-caspase-3 was increased. In contrast, the expression of Bax was suppressed, suggesting its positive effects in attenuating oxidative stress and inflammatory responses and inhibiting hepatocyte apoptosis, thus effectively reducing APAP-induced liver injury.

Hu et al. [28] investigated the effect of DHQ on APAP-induced hepatotoxicity in mice. They found that 80 mg/kg of DHQ alone effectively inhibited the expression of CYP2E1 in the liver tissue of mice. In addition, DHQ reversed the APAP-induced decrease in L-02 cell viability and reduced the accumulation of intracellular ROS levels. Glutamate cysteine ligase (GCL), a key rate-limiting enzyme for synthesizing glutathione, is essential for enhancing the body’s resistance to oxidative stress. GCLC and GCLM are the catalytic and regulatory subunits of GCL, respectively [29,30,31]. Following DHQ treatment, the antioxidant function was achieved by upregulating the expression of GCLC, thereby preventing APAP-induced liver injury.

Zai et al. [32] investigated the protective effect of DHQ against APAP-induced hepatocyte injury with different concentrations (12.5–100 μM). It was discovered that DHQ attenuated APAP-induced cell proliferation inhibition and lactate dehydrogenase (LDH) release in a dose-dependent manner and was able to block APAP-induced cell necrosis and extracellular signal-regulated kinase-c-Jun-N-terminal kinase (ERK-JNK) stress responses. In addition, DHQ improved cellular ROS levels in a dose-dependent manner. Mitochondrial dysfunction is a crucial factor in generating high levels of reactive oxygen species (ROS), and an initial increase in ROS will further exacerbate mitochondrial damage [33]. The results showed that APAP treatment resulted in the loss of mitochondrial membrane potential (MMP), followed by the reversal of ROS accumulation and mitochondrial dysfunction. DHQ also initiated the Janus kinase 2/signal transducer and activator of transcription 3 (JAK2/STAT3) cascade phosphorylation process, thereby regulating the expression of anti-apoptotic Bcl-2 family proteins. In addition, DHQ induces autophagy, mediating its protective effect on hepatocytes. Notably, this protective effect can be reversed by intervening with chloroquine (CQ) and inhibiting the autophagic process.

#### 2.1.2. Ameliorative Effects against Carbon Tetrachloride (CCl_4_)-Induced Liver Injury

During CCl_4_-induced hepatic fibrosis, free radicals and cytokines released from damaged hepatocytes activate HSCs and initiate fibrogenesis [34]. The characteristics of CCl_4_-induced histological and biochemical changes reflect the pattern of human liver fibrosis and cirrhotic disease. In addition, some of the mechanisms of CCl_4_ induction have been demonstrated [35,36].

Liu et al. [37] treated CCl_4_-induced acute liver injury in mice with different concentrations (20, 40, and 80 mg/kg) of DHQ, and the present study demonstrated that DHQ inhibited hepatic stellate cell (HSC) activation and extracellular matrix production by regulating the PI3K/AKT/mTOR and TGF-β1/Smads pathways. Additionally, DHQ was found to attenuate CCl4-induced oxidative stress and apoptosis, suggesting its potential as an effective hepatoprotective agent.

#### 2.1.3. Ameliorative Effects against Other Drug-Induced Liver Injury

Cyclophosphamide (CP), as a potent anticancer drug and immunosuppressant, has demonstrated significant clinical efficacy [38,39,40]. Meanwhile, it is associated with numerous adverse effects, especially causing hepatic impairment [41]. Althunibat et al. [42] investigated the potential protective role of DHQ by a mouse model of CP-induced hepatotoxicity. NF-κB p65 activation and pro-inflammatory cytokines are key factors in CP-induced liver injury. It was shown that DHQ treatment reduced NF-κB p65 protein expression and significantly lowered the levels of TNF-α, IL-1β and IL-6 while effectively inhibiting the process of apoptosis through the up-regulation of the expression of the anti-apoptotic protein Bcl-2 and the reduction in the levels of the pro-apoptotic proteins Bax and caspase-3. Together, these findings support the anti-inflammatory and protective effects of DHQ in attenuating CP-induced liver injury.

As a pesticide ingredient of natural origin, rotenone has been recognized as an environmental pollutant. It has been reported in the literature to cause organ function damage, exhibiting potentially toxic effects [43,44,45]. After using rotenone to cause liver injury, Akinmoladun et al. [46] found that liver function indices after treatment with DHQ (0.25, 0.5, and 1 mg/kg) showed significant improvement, including bilirubin level, γ-glutamyltransferase, alkaline phosphatase, alanine aminotransferase and aspartate aminotransferase activities, and total protein concentration. The levels/activities of hepatic oxidative stress indicators showed that DHQ could effectively attenuate the hepatic injury induced by rotenone and exert its protective effects by enhancing the antioxidant mechanism and reducing the level of oxidative stress.

Pazopanib, a tyrosine kinase inhibitor, is commonly used for the treatment of metastatic renal cell carcinoma and advanced soft tissue sarcoma, and it induces varying degrees of hepatotoxicity [41,47,48]. AkAgunduz et al. [49] explored the effect of DHQ (50 mg/kg) on pazopanib-induced hepatotoxicity. It not only attenuated pazopanib-induced liver injury at the histopathological level but also showed positive improvement in biochemical indexes and effectively inhibited the further development of oxidative hepatotoxicity. These discoveries emphasize the value of DHQ as a potential adjuvant therapy in attenuating the hepatotoxicity of the chemotherapeutic drug pazopanib and provide a theoretical basis for further preclinical and clinical studies.

Cisplatin, a widely used platinum-based chemotherapeutic agent, has been used in the treatment of a wide range of cancer types, including gastric and ovarian cancers [50,51,52]. Although it has demonstrated significant efficacy against these malignant tumors and the efficacy is usually enhanced with the elevated drug dose, its non-selective mechanism of action also poses a severe challenge to patients [53,54]. With increasing therapeutic doses, cisplatin inevitably produces toxic effects on several organs and physiological systems, such as hepatotoxicity and nephrotoxicity, limiting its clinical application [55,56]. Kurt et al. [57] investigated the effect of DHQ on cisplatin-induced oxidative liver injury in rats using biochemical methods. After treatment with DHQ (50 mg/kg), the reduction in serum alanine aminotransferase (AST) and alanine aminotransferase (ALT) levels and the normalization of other biochemical indices were observed. The intervention of DHQ effectively reduced the degree of oxidative stress and thus exerted a protective effect on the liver.

### 2.2. Ameliorative Effects on Alcoholic Liver Injury

**Figure 2:**
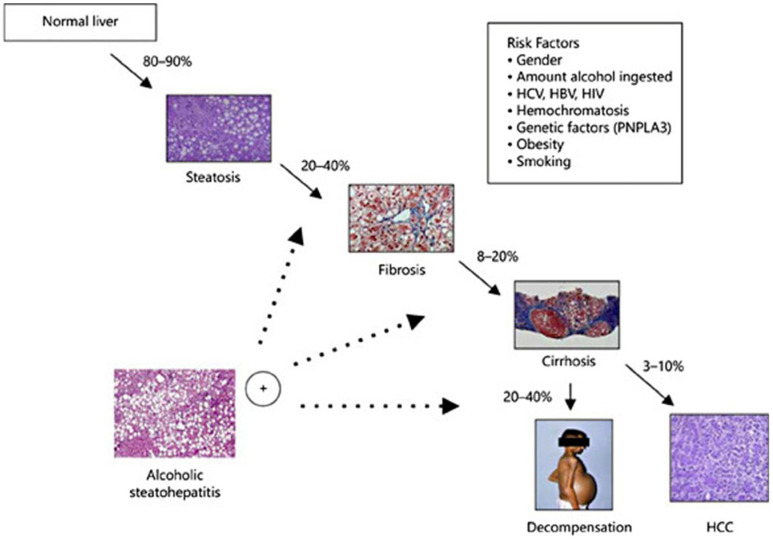
Progression of ALD and susceptible risk factors for accelerated progression [64]. (These figures were reprinted with permission).

**Figure 3:**
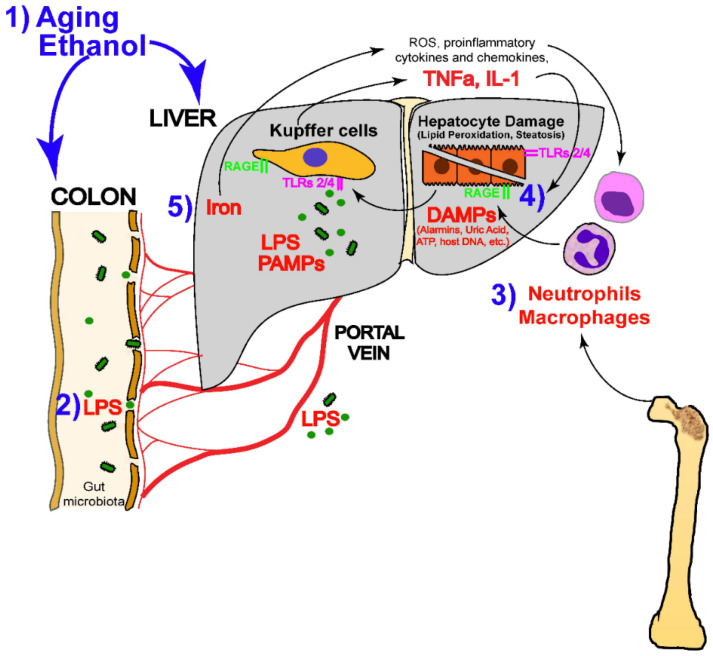
Alcohol-mediated liver injury and the mechanisms that drive it (LPS, lipopolysaccharide; DAMP, disease-associated molecular patterns; ATP, adenosine triphosphate; TNFα, tumor necrosis factor-α; IL-1, interleukin-1; ROS, reactive oxygen species; PAMPs, pathogen-associated molecular patterns) [65]. (These figures were reprinted with permission).

Excessive alcohol consumption has varying chances of leading to the development of different kinds of alcohol-related liver disease (ALD), including a range of diseases such as asymptomatic hepatic steatosis, hepatic fibrosis, and cirrhosis (Figure 2) [58,59,60,61,62]. Alcohol metabolite toxicity reduces liver compensatory capacity, interferes with homeostasis, and makes the liver vulnerable [63]. Critical aspects in the development of ALD include (1) aging and gender differences; (2) aging and gender differences; (3) infiltration of neutrophils and bone marrow-derived macrophages; (4) alcohol metabolism and fatty acid synthesis; and (5) iron deposition and ROS production (Figure 3).

In order to explore the pathogenesis of ALD, interventional therapy, and to understand the effects of alcohol metabolism on the microenvironment of liver tissues, Ding et al. [8] explored the potential protective effects of different concentrations of DHQ (20, 40, and 80 mg/kg) against acute alcoholic liver injury in mice. The experimental results showed that the treatment of DHQ decreased alanine aminotransferase (ALT) and increased aspartate aminotransferase (AST) while contributing to the increase in superoxide dismutase (SOD), glutathione (GSH) and malondialdehyde (MDA). Histopathologic examination showed that alcohol-induced hepatocellular injury and inflammatory invasion were reduced after treatment with DHQ. These findings support the role of DHQ as a hepatoprotective agent, which contributes to the amelioration of alcoholic liver injury through its antioxidant, anti-inflammatory, and direct protective effects on hepatocytes. Meanwhile, Western blot analysis and real-time fluorescence quantitative PCR (rt-PCR) results showed that DHQ was able to reduce the level of tumor necrosis factor-α (TNF-α), block the activation pathway of nuclear factor-κB (NF-κB) in the liver, and effectively reverse the alcohol-induced. The process of alcohol-induced apoptosis was effectively reversed by adjusting the expression of PI3K/Akt signaling pathway and its downstream apoptosis-related factors.

### 2.3. Ameliorative Effects on Fatty Liver Injury

**Figure 4:**
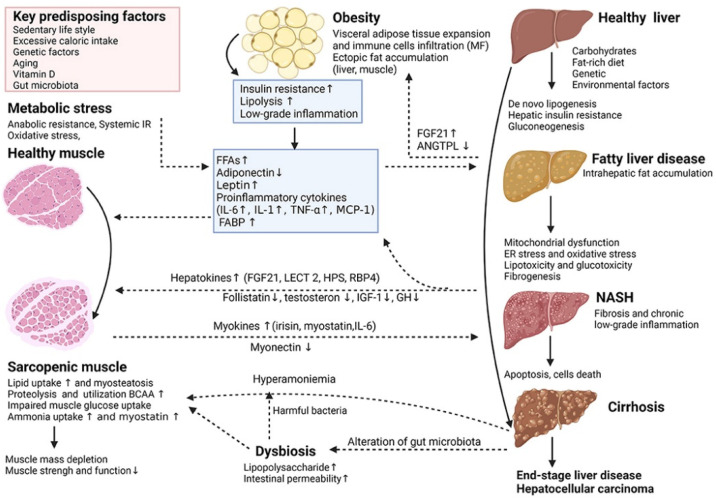
Key cellular and molecular mechanisms underlying the complex interactions between adipose tissue, sarcopenia and non-alcoholic fatty liver disease (NAFLD) [72]. (These figures were reprinted with permission).

Non-alcoholic fatty liver disease (NAFLD) is one of the most prevalent types of chronic liver disease worldwide, affecting approximately one-quarter of the general population, with a particular predilection for patients with obesity and type 2 diabetes mellitus (T2DM) [66,67,68]. The scope of the disease is broad, encompassing both non-alcoholic simple fatty liver disease (NAFL) and the more severe non-alcoholic steatohepatitis (NASH). Notably, patients with NASH have a significantly higher risk of developing advanced liver fibrosis, cirrhosis, and even hepatocellular carcinoma (HCC) [69,70,71]. The pathomechanism of NASH is complex (Figure 4), involving the interplay of metabolic stress and inflammatory response. Given this, therapeutic strategies for NASH need to focus on stopping the progression of the condition to hepatocellular carcinoma, and it is therefore critical to assess whether therapies are effective in blocking this process.

Lee et al. [73] explored the effect of DHQ on free fatty acid (FFA)-induced insulin resistance in the liver. FFA treatment inhibited cellular glucose uptake, whereas DHQ at concentrations of 25 and 50 µM was able to reverse this effect and promote glucose uptake. In addition, DHQ upregulated the expression levels of key proteins involved in insulin signaling—p-PI3K, p-IRS1, p-AKT, p-AMPK and p-ACC—in FFA-treated hepatocytes. It was also noted that FFA treatment led to a rise in miR-195 expression, but DHQ treatment significantly reduced it dose-dependently. The modulation of miR-195 levels by the transfection of miR-195 mimics and inhibitors, respectively, further confirmed that DHQ enhanced the expression of p-IRS1, p-PI3K, p-AMPK, p-AKT, and p-ACC through this pathway, thus revealing the mechanism by which DHQ alleviates FFA-induced liver damage by modulating miR-195 expression. These findings provide new perspectives for understanding the molecular mechanisms of DHQ in ameliorating fatty liver-associated insulin resistance.

Inoue et al. [74] DHQ at different concentrations (0.05 and 3%) used in the treatment of a NASH mouse model significantly prevented the development of hepatic steatosis, chronic inflammation and liver fibrosis. The mechanism of action includes a direct effect on hepatocytes, i.e., the inhibition of lipid accumulation [75]. FGF21 is a potent inducer of thermogenic genes in brown adipose tissue [76]. mRNA expression of FGF21 was increased in the liver and brown adipose tissue by DHQ treatment and significantly increased brown adipocyte-specific genes, and mature brown adipocyte secreted factors (FGF21 and IL-6) of mRNA expression. These results suggest that DHQ acts through a dual mechanism: directly regulating brown adipocyte function while promoting FGF21 expression in the liver. When exploring the effects of DHQ on hepatic steatosis in depth, the researchers found that the up-regulated expression of genes associated with lipogenesis (SREBP1c, FAS, SCDc1, and ACC and inflammatory genes (TNFα, Il-1β, and EMR1 (F4/80)) was significantly suppressed in the high-dose DQH-treated group. The histological examination further revealed the presence of small, dysplastic nodules characterized by atypical hepatocellular hyperplasia with enlarged nuclei and deepened pigmentation in non-tumorigenic NASH liver samples. DHQ treatment significantly reduced the mRNA expression of genes associated with inflammation and fibrosis in liver tumor lesions without affecting CD206, a representative marker of tumor-associated macrophages. In summary, the study by Inoue et al. not only provides strong evidence for the therapeutic effects of DHQ in successive stages of NASH but also provides insight into the novel mechanisms of DHQ action.

### 2.4. Ameliorative Effects against Autoimmune Hepatitis

Autoimmune hepatitis is a severe health challenge affecting a large number of patients worldwide. The cases of the disease increased abruptly in 2014 (Table 1), and disease progression is often accompanied by a poor prognosis [77]. In this context, DHQ, through its potent antioxidant properties and anti-inflammatory effects, can significantly alleviate the symptoms of acute or severe (fulminant) hepatitis, offering new hope for intervening in this type of autoimmune liver disease [78]. Zhao et al. [79] found that DHQ at a dose of 30 mg/kg significantly ameliorated cutin A (Con A)-induced liver injury in mice. Specifically, DHQ was able to significantly enhance the survival rate of mice while effectively reducing the serum levels of alanine aminotransferase (ALT) and aspartate aminotransferase (AST), indicators of liver function. Given that oxidative stress and pro-inflammatory mediators released by macrophages play a vital role in the development of immune-mediated hepatitis [80], DHQ demonstrated the ability to significantly inhibit interferon-gamma (IFN-γ) and tumor necrosis factor-α (TNF-α) mRNA expression and their secretion. This mechanism of action involves the enhancement of nuclear factor E2-related factor 2 (Nrf2) expression, which is not only increased in the cytoplasm but also shifted to the nucleus, thereby significantly elevating heme oxygenase-1 (HO-1) expression in a time- and dose-dependent manner. The protective effect of DHQ on Con A-induced hepatic injury may be due to the activation of Nrf2/1. The protective mechanism of DHQ against Con A-induced liver injury may lie in the activation of the Nrf2/HO-1 pathway to remove oxidative stress and increase HO-1 activity, as well as the modulation of MAPK signaling in macrophages to inhibit the release of inflammatory mediators, thereby effectively alleviating immune-mediated liver injury. Together, these findings emphasize the effectiveness and mechanism of DHQ as a potential therapeutic tool in response to specific models of liver injury.

Chen et al. [81] identified that the immunomodulatory effects of DHQ on DHQ (5 mg/kg) effectively ameliorated Con A-mediated immune, hepatic injury by reducing the expression of pro-inflammatory mediators and inhibiting the infiltration of CD4+/CD8+ T cells in the liver. In addition, DHQ may also provide HepG2 cells with protective effects against TNF-α/ActD-induced apoptosis by modulating the caspase pathway and NF-κB signaling pathway, thus demonstrating its dual benefits in immunomodulation and cytoprotection.

**Table 1:** Studies on the incidence and prevalence of autoimmune hepatitis [82]. (The data are reprinted with permission).

Incidence/100,000	Rrevalence/100,000	Ref.	Year	Cases
0.8	——	[83]	1997	496
3.0	——	[84]	2007	200
0.85	10.7	[85]	2008	473
1.68	23.9	[78]	2014	1721
1.1	18.3	[86]	2014	1313
2.0	24.5	[87]	2010	138
0.67	11.0	[88]	2013	100
1.37	11.61	[89]	2004	13
——	42.9	[90]	2002	77

## 3. Improvement of DHQ Bioavailability

**Figure 5:**
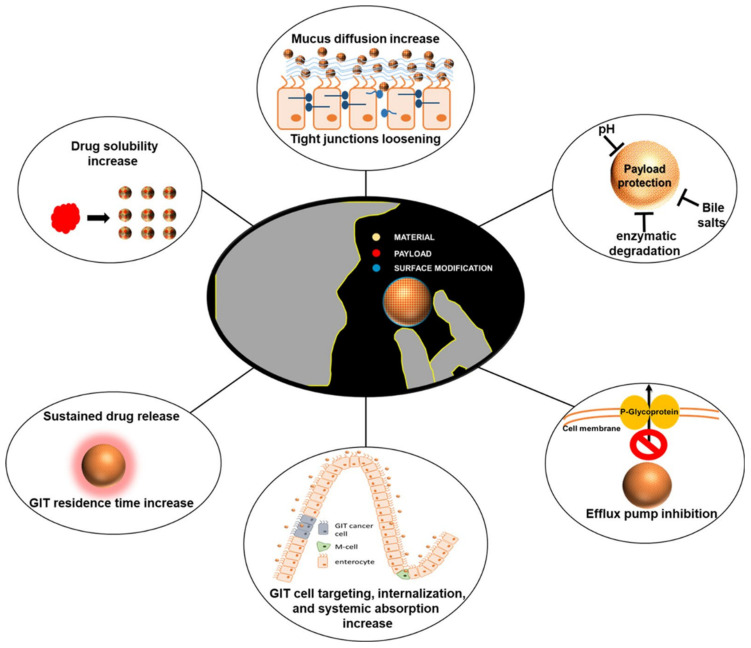
Characterization of nanodelivery systems for improving the bioavailability of oral therapeutic drugs [103]. (These figures were reprinted with permission).

Drug bioavailability is an essential aspect of pharmacology that affects the effectiveness of drug therapy [91]. Although a large number of studies have amply demonstrated the great potential of DHQ for clinical applications, in fact, systematic studies on the stability of natural products are quite limited [92]. Previous studies have pointed out that DHQ undergoes polymerization when electrolyzed in a neutral environment (pH 7.0) [93], which undoubtedly poses a challenge to the stable use of the drug. In addition, despite its high water solubility, the oral bioavailability of DHQ is unsatisfactory, being only about 36%, especially in the form of lipid-based drug delivery [94,95]. Of more significant concern, DHQ may also be broken down by microbial communities in the gut [96], further affecting its bioavailability. Given all these limitations, finding effective methods to enhance the bioavailability of DHQ has become a key aspect in driving the development of novel drug delivery systems. This requires an in-depth investigation of the chemical and biological properties of DHQ, but also innovative drug formulation techniques to ensure that the compound is absorbed and utilized more stably and efficiently after entry into the body, thus fully exploiting its potential therapeutic benefits (Figure 5).

The development of innovative materials is leading a revolution in medicine, significantly advancing the progress of drug manufacturing [97]. Xiong and other scholars [98] have exhaustively elaborated in their study a cutting-edge strategy that utilizes metal-organic frameworks (MOFs) cross-linked with cyclodextrins to significantly enhance the efficiency of chemotherapeutic drug-targeted delivery and uptake to localized lesions. This technological breakthrough opens up new possibilities for precision medicine. In another study, Song et al. [99] introduced a novel class of organic mineral matrix materials, such as liposomal dispersions prepared using natural clay. These novel materials not only ensured the stability of the formulation but also broadened the solubility range of the drug, providing an innovative solution for the delivery of difficult-to-solve drugs. Ding et al. [100] explored the hepatoprotective mechanism of DHQ liposomes by in vivo experiments after preparing DHQ liposomes using a thin-film dispersion method. The results showed that DHQ liposomes (50 mg/kg) significantly inhibited LPS/D-galactose-induced acute hepatic injury in mice through antioxidant effects. A review article by V. Ambrogi [101], on the other hand, looked at the functional expansion of traditional materials from an entirely new perspective. Taking calcium carbonate as an example, this classic material, which has been traditionally used as pH-responsive fillers, is now being utilized and revitalized through the exploration of the application of different crystal forms. Calcium carbonate is being developed as a novel carrier matrix for drug molecules [102], and utilizing its unique physical and chemical properties, these matrices can effectively enhance the bioavailability of drugs, bringing revolutionary thinking to drug design and delivery technologies. These recent advances in material science undoubtedly provide strong support for improving drug therapeutic efficacy, reducing side effects and developing novel drug release systems.

## 4. Prospects and Outlook

**Figure 6:**
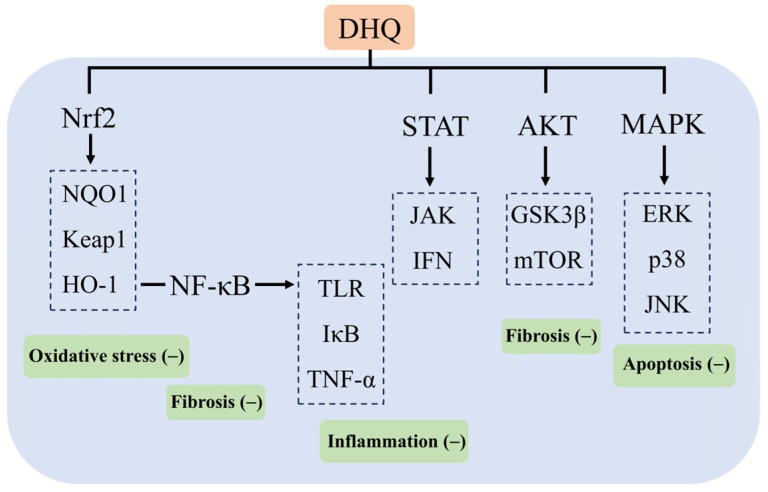
Mechanism of action of DHQ in ameliorating liver injury.

In Russia, DHQ is added to more than 250 products, of which 142 are food supplements and 40 are food products, and no adverse reactions have been reported during the 15–20 years of use and sale of their products [104]. DHQ has been included in the Russian Pharmacopoeia for hepatoprotection, the treatment of diabetes, atherosclerosis, and so on. Some patients have been taking 600 mg per day for two weeks, with no adverse reactions. Meanwhile, on 13 December 2016, a panel of experts from the European Union Food Safety Authority EFSA reviewed the composition of the DHQ extract provided by Ametis JSC. It stated that it was adequately characterized and had no safety concerns, and no genotoxicity was found. A 90-day subchronic rat study according to OECD standards showed no adverse effects at the highest dose (1500 mg/kg body weight) [99,105]. Thus, the aim of this paper is to review the mechanism of action of the natural flavonoid DHQ in the prevention and treatment of various experimental liver injuries and its recent findings. As a crucial organ in the human body, the liver undertakes the core functions of synthesis, metabolism and detoxification. Therefore, the effective prevention and treatment of liver injury have been a hot issue in the field of medical research. The review shows that DHQ is particularly effective in combating chemical, pathological, immune, and pharmacological liver injuries and that DHQ exerts its effects in protecting the liver from injury through a variety of mechanisms, including the attenuation of oxidative stress and inflammation, the modulation of lipid metabolism, antiviral activity, and immunomodulation. These mechanisms of action involve multiple key signaling pathways and their targets, such as TLR and IκB in the NF-κB signaling pathway; ERK, p38, and JNK in the MAPK pathway; GSK3β and mTOR in the Akt pathway; HO-1, NQO1, and Keap1 in the Nrf2 pathway; and JAK and IFN in the STAT pathway, which demonstrates the multidimensional protective effects of DHQ at the molecular level (Figure 6). Although DHQ has demonstrated a wide range of potential applications in experimental studies for the prevention and treatment of liver injury, its low oral bioavailability has become a significant bottleneck limiting its clinical application. For this reason, several strategies to enhance the bioavailability of DHQ are also discussed in the paper, aiming to provide theoretical and technical support for the subsequent DHQ-based clinical trial studies and to promote the conversion of pharmaceutical preparations containing DHQ as the main active ingredient for clinical treatment. In the future, by profoundly exploring the potential clinical value of DHQ in different types of liver injury, as well as further clarifying the specific targets of its action, we can not only lay a solid foundation for the clinical application of DHQ but also provide valuable insights and directions for the development of novel therapeutic drugs for liver diseases, which is expected to make breakthroughs in the field of preventing and treating liver diseases.

## Data Availability

Data supporting the findings are available from the corresponding authors upon reasonable request.
